# N-butanol extracts of Morinda citrifolia suppress advanced glycation end products (AGE)-induced inflammatory reactions in endothelial cells through its anti-oxidative properties

**DOI:** 10.1186/s12906-017-1641-3

**Published:** 2017-03-04

**Authors:** Yuji Ishibashi, Takanori Matsui, Fumiyuki Isami, Yumi Abe, Tatsuya Sakaguchi, Yuichiro Higashimoto, Sho-ichi Yamagishi

**Affiliations:** 1 Department of Pathophysiology and Therapeutics of Diabetic Vascular Complications, Kurume University School of Medicine, Kurume, 830-0011 Japan; 2 Morinda Worldwide Inc., Tokyo, 160-0023 Japan; 3 Department of Chemistry, Kurume University School of Medicine, Kurume, 830-0011 Japan

**Keywords:** AGEs, RAGE, Oxidative stress, Atherosclerosis

## Abstract

**Background:**

Advanced glycation end products (AGEs), senescent macroprotein derivatives formed during a normal aging process and acceleratedly under diabetic conditions, play a role in atherosclerotic cardiovascular disease. AGEs cause endothelial cell (EC) damage, an initial trigger for atherosclerosis through the interaction with a receptor for AGEs (RAGE). We have previously shown that n-butanol extracts of Morinda citrifolia (noni), a plant belonging to the family Rubiaceae, block the binding of AGEs to RAGE in vitro. In this study, we examined the effects of n-butanol extracts of noni on reactive oxygen species (ROS) generation and inflammatory reactions on AGE-exposed human umbilical vein ECs (HUVECs).

**Methods:**

HUVECs were treated with 100 μg/ml AGE-bovine serum albumin (AGE-BSA) or non-glycated BSA in the presence or absence of 670 ng/ml n-butanol extracts of noni for 4 h. Then ROS generation and inflammatory and gene expression in HUVECs were evaluated by dihydroethidium staining and real-time reverse transcription-polymerase chain reaction analyses, respectively. THP-1 cell adhesion to HUVECs was measured after 2-day incubation of AGE-BSA or BSA in the presence or absence of 670 ng/ml n-butanol extracts of noni.

**Results:**

N-butanol extracts of noni at 670 ng/ml significantly inhibited the AGE-induced ROS generation and RAGE, intercellular adhesion molecule-1 and plasminogen activator inhibitor-1 gene expressions in HUVECs. AGEs significantly increased monocytic THP-1 cell adhesion to HUVECs, which was also prevented by 670 ng/ml n-butanol extracts of noni.

**Conclusions:**

The present study demonstrated for the first time that N-butanol extracts of noni could suppress the AGE-induced inflammatory reactions in HUVECs through its anti-oxidative properties via blocking of the interaction of AGEs with RAGE. Inhibition of the AGE-RAGE axis by n-butanol extracts of noni may be a novel nutraceutical strategy for the treatment of cardiovascular disease.

## Background

Reactive derivatives from non-enzymatic reactions between sugars and amino groups of proteins, lipids and nucleic acids form a heterogeneous group of irreversible adducts called “AGEs (advanced glycation end products)” [1–3]. The formation and accumulation of AGEs have been known to progress at a physiological normal aging process and more acceleratedly under diabetic conditions, thereby being involved in the development and progression of atherosclerotic cardiovascular disease [1–14]. Indeed, diabetic apolipoprotein E-deficient mice fed an AGE-restricted diet exhibited less atherosclerotic lesions, which were associated with decreased AGEs, receptor for AGEs (RAGE) and inflammatory cells in the aortic roots [5]. There was a correlation between AGE levels and the degree of atheroma in cholesterol-fed rabbits, whereas treatment with aminoguanidine, an inhibitor of AGE formation decreased plaque formation in the aortae of these animals [6]. Administration of a recombinant soluble form of RAGE consisting of the extracellular AGE-binding domain, has not only suppressed the development of atherosclerosis but also stabilized established atherosclerosis in diabetic apolipoprotein E-null mice [7, 8]. RAGE-deficient mice were found to be resistant to the development of atherosclerosis when they were rendered diabetic [9]. Furthermore, circulating levels of AGEs have also been shown to predict total and cardiovascular disease mortality in both type 1 and type 2 diabetic patients [10, 11]. These observations suggest that the inhibition of the AGE-RAGE axis is a novel therapeutic target for atherosclerotic cardiovascular disease, espeicially in elderly people or diabetic patients.

We have previously shown that n-butanol extracts of Morinda citrifolia (noni), a plant belonging to the family Rubiaceae, which has been used for centuries by Pacific Islanders as an alternative medicine for various disorders, such as diabetes and arthritis [15, 16], block the binding of AGEs to RAGE in vitro [17]. In this study, we examined the effects of n-butanol extracts of noni on reactive oxygen species (ROS) generation and inflammatory reactions on AGE-exposed human umbilical vein endothelial cells (HUVECs).

## Methods

### Materials

Bovine serum albumin (BSA) (essentially fatty acid free and essentially globulin free, lyophilized powder) and D-glyceraldehyde were purchased from Sigma (St. Louis, MO, USA). Ethyl acetate and n-butanol were purchased from Wako Pure Chemical Industries, Ltd. (Osaka, Japan).

### Preparation of AGE-BSA

AGE-modified BSA was prepared as described previously [18]. In brief, BSA (25 mg/ml) was incubated under sterile conditions with 0.1 M glyceraldehyde in 0.2 M NaPO_4_ buffer (pH 7.4) at 37 °C for 7 days. Control non-glycated BSA was incubated in the same conditions except for the absence of glyceraldehyde as described previously [18].

### Cells

HUVECs obtained from Lonza Group Ltd. (Basel, Switzerland) were cultured in endothelial basal medium supplemented with 2% fetal bovine serum, 0.4% bovine brain extracts, 10 ng/ml human epidermal growth factor and 1 μg/ml hydrocortisone according to the manufacturer’s recommendation. AGE or non-glycated BSA treatment was carried out in a medium lacking epidermal growth factor and hydrocortisone. Cells at passage 4–11 were used for the present experiments. According to the certificate of analysis by Lonza Group Ltd., HUVECs were pooled from Caucasian newborn babies. Isolated cells were identified as HUVECs because cells expressed CD31/105, von Williebrand Factor VIII, and were positive for acetyated low-density lipoprotein uptake. All cells were negative for mycoplasma.

### Preparation of n-butanol extracts of noni

Morinda citrifolia fruits were collected in French Polynesia during 2004–2006 and identified by botanists at Tropical Resources, Inc. (Provo, UT, USA) where voucher specimens were deposited (Part No. 119107, Lot Code 52488, Batch # 23401). The fruits were separated into flesh and seeds by hand. The flesh was freeze-dried, extracted with 50% ethanol, and then the extracts were further extracted with ethyl acetate and n-butanol several times as described previously [19].

### Dihydroethidium (DHE) staining

HUVECs were treated with 100 μg/ml AGE-BSA or non-glycated BSA in the presence or absence of 670 ng/ml n-butanol extracts of noni for 4 h. Then the cells were incubated with phenol red free Dulbecco's Modified Eagle Medium containing 3 μM DHE (Molecular Probes Inc., Eugene, OR, USA). After 15 min, ROS generation was evaluated by intensity of DHE staining as described previously [20]. Intensity of DHE staining in five different field of each sample was analyzed by microcomputer-assisted image J.

### Real-time reverse transcription-polymerase chain reactions (RT-PCR)

HUVECs were treated with 100 μg/ml AGE-BSA or non-glycated BSA in the presence or absence of 670 ng/ml n-butanol extracts of noni for 4 h. Then total RNA was extracted with RNAqueous-4PCR kit, and quantitative real-time RT-PCR was performed using Assay-on-Demand and TaqMan 5 fluorogenic nuclease chemistry (Applied Biosystems, Foster city, CA, USA) as described previously [20]. IDs of primers for human RAGE, intercellular adhesion molecule-1 (ICAM-1), plasminogen activator inhibitor-1 (PAI-1), β-actin, and 18S gene were Hs00542592_g1, Hs00164932_m1, Hs01126606_m1, Hs01060665_g1, and Hs03003631_g1, respectively.

### Assay of THP-1 cell adhesion to HUVECs

Human THP-1 monocytic leukemia cells were purchased from American Type Culture Collection (Manassas, VA, USA) and labeled with 3 μM BCECF-AM (Dojindo, Kumamoto, Japan) as described previously [21]. HUVECs were treated with 100 μg/ml AGE-BSA or non-glycated BSA in the presence or absence of 670 ng/ml n-butanol extracts of noni for 24 h and then incubated with BCECF-AM-labeled THP-1 cells for 4 h. After the incubation, non-adherent THP-1 cells were removed, and fluorescent intensities of the adherent THP-1 cells were measured as described previously [21].

### Statistical analysis

All values were presented as mean ± standard error. One-way ANOVA followed by Student’s *t*-test was performed for statistical comparisons; *p* < 0.05 was considered significant. All statistical analyses were performed using the R version 3.2.5 (Copenhagen Business School, Frederiksberg, Denmark).

## Results

### Effects of noni extracts on ROS generation

**Fig. 1:**
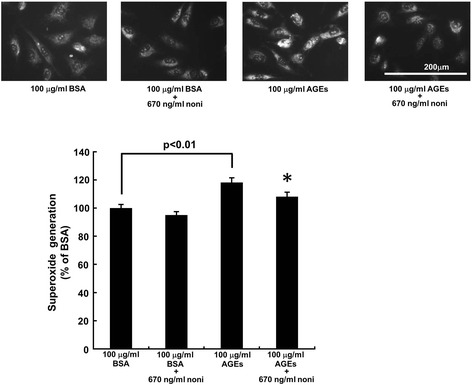
Effects of noni extracts on ROS generation in AGE-exposed HUVECs. HUVECs were treated with 100 μg/ml AGE-BSA or non-glycated BSA in the presence or absence of 670 ng/ml n-butanol extracts of noni for 4 h. Then superoxide generation was evaluated by intensity of DHE staining. *Upper panels* show the representative photos of DHE stainings. *Lower panel* shows the quantitative data. *N* = 3 per group. *, *p* < 0.05 compared to the value with AGEs alone

We first examined the effects of n-butanol extracts of noni on ROS generation in HUVECs. As shown in Fig. 1, AGEs significantly increased ROS generation in HUVECs, which was prevented by 670 ng/ml n-butanol extracts of noni. Noni extracts did not affect ROS generation in non-glycated BSA-exposed HUVECs.

### Effects of noni extracts on RAGE, ICAM-1 and PAI-1 gene expressions

**Fig. 2:**
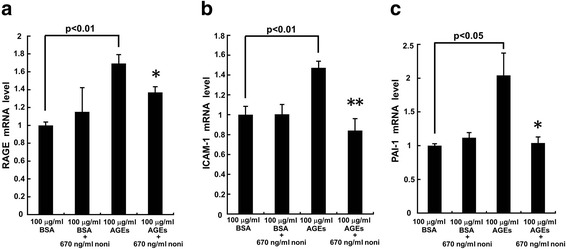
Effects of noni extracts on RAGE (**a**), ICAM-1 (**b**), and PAI-1 (**c**) mRNA levels in AGE-exposed HUVECs. HUVECs were treated with 100 μg/ml AGE-BSA or non-glycated BSA in the presence or absence of 670 ng/ml n-butanol extracts of noni for 4 h. Then total RNAs were transcribed and amplified by real-time PCR. Data were normalized by the intensity of β-actin (**a**) or 18S mRNA-derived signals (**b** and **c**) and then related to the value obtained with non-glycated BSA treatment alone. **a** *N* = 4 per group. **b** and **c** *N* = 8 per group. * and **, *p* < 0.05 and *p* < 0.01 compared to the value with AGEs alone, respectively

We next investigated the effects of noni extracts on inflammatory reactions in HUVECs. AGEs up-regulated RAGE mRNA levels in HUVECs, which was associated with the increases in ICAM-1 and PAI-1 gene expressions (Fig. 2). N-butanol extracts of noni at 670 ng/ml significantly blocked the AGE-induced gene expressions of RAGE, ICAM-1 and PAI-1 in HUVECs. Noni extracts did not affect these mRNA levels in non-glycated BSA-exposed HUVECs.

### Effects of noni extracts on THP-1 cell adhesion to HUVECs

**Fig. 3:**
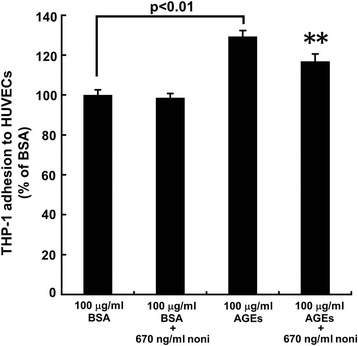
Effects of noni extracts on THP-1 cell adhesion to AGE-exposed HUVECs. HUVECs were treated with 100 μg/ml AGE-BSA or non-glycated BSA in the presence or absence of 670 ng/ml n-butanol extracts of noni for 24 h. Then HUVECs were incubated with BCECF-AM-labeled THP-1 cells for 4 h. Non-adherent THP-1 cells were removed. Fluorescent intensities of the adherent THP-1 cells were measured. *N* = 6 per group. **, *p* < 0.01 compared to the value with AGEs alone

We further studied the effects of noni extracts on THP-1 cell adhesion to HUVECs. As shown in Fig. 3, 670 ng/ml n-butanol extracts of noni significantly prevented the increase in THP-1 cell adhesion to AGE-exposed HUVECs. Noni extracts did not affect THP-1 cell adhesion to non-glycated BSA-exposed HUVECs.

## Discussion

There is an accumulating body of experimental and epidemiological evidence that atherosclerosis is intrinsically an inflammatory disease and that endothelial cell damage and dysfunction are an initial event that leads to the development of atherosclerotic cardiovascular disease [22–24]. Furthermore, recent understandings of the disease process have also revealed that AGEs and RAGE interaction could evoke oxidative stress generation and cause inflammatory reactions, thus contributing to the increased risk for cardiovascular disease in elderly patients, especially poorly controlled diabetes [4–9, 25, 26]. We have very recently found that n-butanol extracts of noni could prevent the interaction of AGEs with RAGE in vitro by binding RAGE with a dissociation constant of 640 μg/ml and a half maximal inhibitory concentration of 200 ng/ml [17]. Therefore, we examined here the effects of n-butanol extracts of noni on the AGE-RAGE axis in HUVECs.

In this study, we found for the first time that n-butanol extracts of noni significantly inhibited the AGE-induced ROS generation, RAGE, ICAM-1 and PAI-1 gene expressions in HUVECs, which were associated with attenuation of THP-1 cell adhesion to AGE-exposed HUVECs. We have previously shown that antibody or antisense DNA raised against RAGE completely inhibits the AGE-evoked ROS generation in endothelial cells, while an anti-oxidant *N*-acetylcysteine or RAGE antibody itself blocks up-regulation of RAGE mRNA levels in AGE-exposed endothelial cells [21, 27, 28]. So our present study suggests that n-butanol extracts of noni may inhibit the deleterious effects of AGEs by breaking the vicious cycle between ROS generation and RAGE overexpression in HUVECs.

ICAM-1 stimulates the recruitment and firm adhesion of inflammatory cells to endothelial cells, which could promote inflammatory reactions in atherosclerosis [29]. Furthermore, attenuated fibrinolytic activity by increased PAI-1 levels is associated with the increased risk for atherothrombosis and cardiovascular disease in diabetic patients [30]. Given that AGEs could induce ICAM-1 and PAI-1 gene expressions in HUVECs via ROS generation through the interaction with RAGE [21, 31], blockade of the AGE-RAGE interaction by n-butanol extracts of noni may be a central mechanism by which they could protect against AGE-induced endothelial cell injury, thus becoming a therapeutic target for atherosclerotic cardiovascular disease. Iridoids are found in various types of medicinal plants, including noni, have been reported to block the inflammatory reactions in AGE-exposed mesangial cells and kidneys of diabetic mice [32, 33]. It would be interesting to examine whether iridoids could actually inhibit the binding of AGEs to RAGE in vitro.

### Limitations

Although the composition of noni fruits has been extensively studied and there are a lot of data available on how to analyze the bioactive compounds [15–17, 19], the active substance has not been fully characterized in the present study. Further experiments using high performance liquid chromatography would be helpful to address the issue.

In this study, freeze-dried flesh of Morinda citrifolia was extracted first with 50% ethanol, and then with ethyl acetate and n-butanol several times. The reason why the native ethanol extracts were not included in this study was that they were inactive; their binding affinity to RAGE was less than one-sixth of those of n-butanol extracts (data not shown). Furthermore, n-butanol extracts alone did not affect superoxide generation or inflammatory reactions in BSA-exposed HUVECs (Figs. 2 and 3). So it is unlikely that the solvents exerted toxic effects on HUVECs. However, the relevance of n-butanol extracts as a potential therapeutic agent in diabetic vascular complications remains unclear.

We used freeze-dried samples after more than 10 years of storage in the present experiments. However, just after the collection during 2004–2006, the fruits were freeze-dried and stored in dark place at 4 °C. Our previous and present studies have suggested that the butanol extracts of freeze-dried and stored samples are bioactive because they not only inhibited the binding of AGEs to RAGE [17], but also suppressed the harmful effects of AGEs on HUVECs. It would be interesting to compare the bioactivity of fresh samples with that of stored samples in HUVECs.

In the present study, the effects of AGEs on ICAM-1 and PAI-1 mRNA levels were much stronger than on RAGE mRNA levels. Although AGEs have been shown to evoke the inflammatory reactions in HUVECs via RAGE interaction [21, 31], the time-response curves of gene expressions may differ.

## Conclusions

The present study suggests that n-butanol extracts of noni may inhibit inflammatory reactions in AGE-exposed HUVECs through its anti-oxidative properties. Since we have found that circulating levels of AGEs are associated with vascular inflammation and endothelial dysfunction in humans [34, 35], blockade by noni extracts of the interaction of AGE with RAGE may be a novel therapeutic strategy for atherosclerotic cardiovascular disease.

## Abbreviations

AGEs: Advanced glycation end products
BSA: Bovine serum albumin
DHE: Dihydroethidium
HUVECs: Human umbilical vein endothelial cells
ICAM-1: Intercellular adhesion molecule-1
PAI-1: Plasminogen activator inhibitor-1
RAGE: Receptor for AGEs
ROS: Reactive oxygen species
RT-PCR: Real-time reverse transcription-polymerase chain reactions
